# Optical properties of Ni and Cu nanowire arrays and Ni/Cu superlattice nanowire arrays

**DOI:** 10.1186/1556-276X-7-569

**Published:** 2012-10-15

**Authors:** Yaya Zhang, Wen Xu, Shaohui Xu, Guangtao Fei, Yiming Xiao, Jiaguang Hu

**Affiliations:** 1 Department of Physics, Yunnan University, Kunming, 650091, China; 2 Key Laboratory of Materials Physics, Institute of Solid State Physics, Chinese Academy of Sciences, Hefei, 230031, China; 3 Department of Math and Physics, , Wenshan, 663000, China

**Keywords:** Nanowire array, Optical properties, Visible and near-infrared, Temperature dependence

## Abstract

In this study, Ni and Cu nanowire arrays and Ni/Cu superlattice nanowire arrays are fabricated using standard techniques such as electrochemical deposition of metals into porous anodic alumina oxide templates having pore diameters of about 50 nm. We perform optical measurements on these nanowire array structures. Optical reflectance (OR) of the as-prepared samples is recorded using an imaging spectrometer in the wavelength range from 400 to 2,000 nm (i.e., from visible to near-infrared bandwidth). The measurements are carried out at temperatures set to be 4.2, 70, 150, and 200 K and at room temperature. We find that the intensity of the OR spectrum for nanowire arrays depends strongly on the temperature. The strongest OR can be observed at about *T* = 200 K for all samples in visible regime. The OR spectra for these samples show different features in the visible and near-infrared bandwidths. We discuss the physical mechanisms responsible for these interesting experimental findings. This study is relevant to the application of metal nanowire arrays as optical and optoelectronic devices.

## Background

In recent years, quasi one-dimensional (1D) nanostructured materials have received much attention attributed to their interesting physical properties in sharp contrast to the bulk ones and to the potential applications as electronic, magnetic, photonic, and optoelectronic devices [1-4]. From a viewpoint of physics, the basic physical properties of nanostructured materials differ significantly from those of bulk materials with the same chemical components. In particular, quantum confinement effects can be observed in the dimensionally reduced nanomaterial systems. Therefore, nanowires have been a major focus of research on nanoscaled materials which can be taken as a fundamental building block of nanotechnology and practical nanodevices. It should be noticed that metal nanowires have displayed unique optical and optoelectronic properties due to surface plasmon resonance (SPR) which is a resonant oscillation of the conducting electrons within the metallic nanostructures. The SPR effect in nanowire structures can cause a tremendous enhancement of the electromagnetic near-field in the immediate vicinity of the particles and can give rise to enhanced scattering and absorption of light radiation. The SPR in metal nanowires and related phenomena (such as the surface-enhanced Raman spectroscopy, nonlinear optic response, plasmonic excitation, to mention but a few) contributes greatly to their promising applications in biosensors, optical devices, and photonic and plasmonic devices [5-8]. Moreover, metal nanowire wave guides can excite and emit terahertz (10^12^ Hz or THz) surface plasmon polaritons [9], which can fill the gap of terahertz electronics and optoelectronics. On the other hand, superlattice nanowires have even richer physical properties owing to further quantum confinement of electron motion along the wire direction. They have been proposed as advanced electronic device systems to observe novel effects such as giant magnetoresistance and even high thermoelectric figure of merit [10,11].

Furthermore, with the rapid development of nanotechnology, it is now possible to fabricate nanowire arrays and superlattice nanowire arrays [12,13]. One of the major advantages to apply nanowire arrays and superlattice nanowire arrays as optic and optoelectronic devices is that the optical response of the array structures can be tuned and modulated via varying sample parameters such as the diameter of the wire and the pattern of the array structure. Due to potential applications of the nanowire arrays and superlattice nanowire arrays as optical devices, it is of importance and significance to examine their basic optical properties. In this article, we present a detailed experimental study on the optical properties of three kinds of nanowire array structures such as Ni and Cu nanowire arrays and Ni/Cu superlattice nanowire arrays. We would like to examine how these advanced nanostructured material systems can respond to light radiation, how their optical properties depend on temperature and radiation wavelength, and why the optical properties of the nanowire arrays differ from those observed in bulk materials.

## Methods

### Samples and measurements

**Figure 1:**
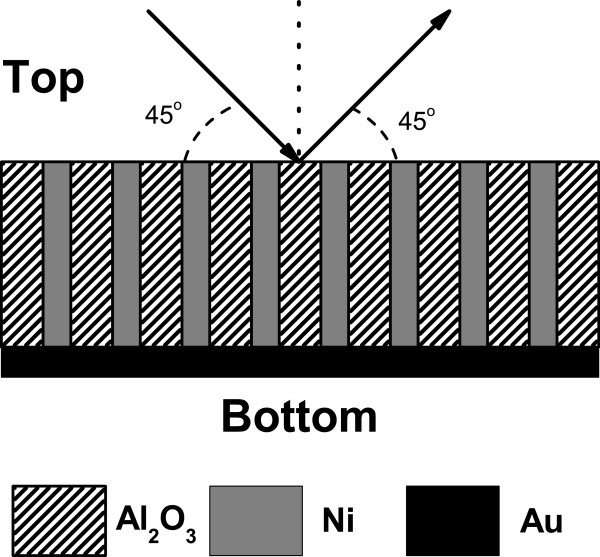
**Schematic diagram of Ni or Cu nanowire arrays in porous anodic alumina membrane.** In the measurement of optical reflection spectrum, the incident light is set at a 45° angle to the sample surface, and the emergent light beam is also at a 45° angle to the sample surface.

In this study, three kinds of nanowire array structures are fabricated, including Ni arrays, Cu arrays, and Ni/Cu superlattice arrays. Samples are prepared by direct current electrodeposition [14-16] of metal into the holes of porous anodic alumina membrane (PAAM) with the pore size of about 50 nm. Noteworthy is the diameter of the nanowires used in the investigation, which is about 50 nm. The length of the nanowires is about 30 *μ*m. The holes of the PAAM are periodically in hexagonal pattern, which can serve as template. The distance between adjacent wires is about 60 nm. Because of the confinement of the PAAM material, metal nanowires grow only along the direction of nanopores of the PAAM template and, therefore, form an array structure. In these samples, a layer of Au film (about 200-nm thick) is sputtered onto one side of the PAAM template to serve as the working electrode. The schematic diagram of the Ni or Cu nanowire array in the PAAM template is shown in Figure 1. For the fabrication of the Ni/Cu superlattice arrays, Ni and Cu materials are deposited alternately into the PAAM holes. The details of the sample fabrication were documented in [14-16].

For the measurement of optical reflection (OR) spectrum, the incident and emergent light beams are set at an angle of 45° to the sample surface (see Figure 1). The measurements are carried out in the visible (400 to 800 nm in wavelength) and near-infrared (1 to 2 *μ*m in wavelength) bandwidths. The tungsten halogen lamp is taken as a white incident light source for the measurements in the visible bandwidth. The Si carbide rod is employed as broadband infrared incident light source for the measurements in the near-infrared bandwidth. The OR spectrum is recorded using an imaging spectrometer (iHR320 HORIBA Jobin Yvon Inc., Edison, NJ, USA) where the PMT is used for the detection of 400- to 800-nm wavelength regime, and the InGaAs photodetector is employed for the measurement of 1- to 2-*μ*m wavelength regime. For measurements in the visible regime, the temperatures are set at 4.2, 70, 150, and 200 K and at room temperature. The change of temperature is achieved in an Oxford cooling system. The measurements in the near-infrared regime are undertaken at room temperature.

## Results and discussion

**Figure 2:**
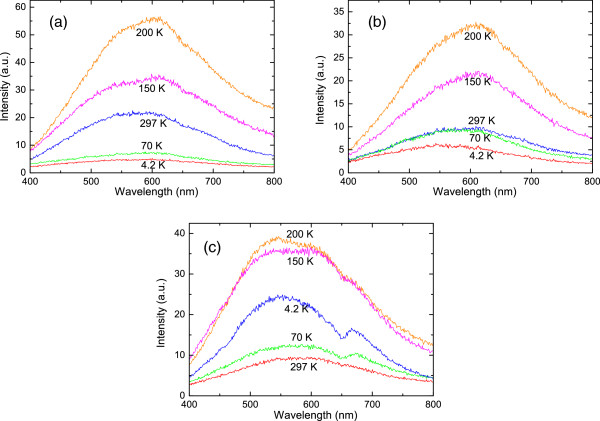
**The spectra of optical reflection for nanowire arrays measured at different temperatures of 4.2, 70, 150, 200, and 297 K as indicated.** The results for a Ni nanowire array (**a**), a Ni/Cu superlattice nanowire array (**b**), and a Cu nanowire array (**c**) are shown.

The OR spectra for Ni and Cu nanowire arrays and Ni/Cu superlattice nanowire arrays are shown in Figure 2 in visible bandwidth for different temperatures at 4.2, 70, 150, 200, and 297 K, respectively. As can be seen, the intensity of OR in nanowire array structures depends strongly on temperature. When temperature (*T*)  < 200 K, the intensity of OR for a Ni nanowire array sample increases with temperature. When *T* > 200 K, the OR intensity decreases with increasing temperature. The strongest OR can be observed at about 200 K. A similar phenomenon can be found for a Ni/Cu superlattice nanowire array sample. In contrast, the OR spectra for Cu nanowire arrays (see Figure 2c) show different temperature dependence. With increasing temperature, the intensity of OR for a Cu nanowire array first decreases in the 4.2- to 70-K regime, then increases in the 70- to 200-K regime, and decreases again when *T* > 200 K. Again, the strongest OR for Cu nanowire arrays can be observed at about *T* = 200 K. These experimental findings suggest that 200 K is an appropriate temperature for the enhancement of optical reflection from Cu, Ni, and Ni/Cu superlattice nanowire array structures. This can provide a basis for further investigation into other optical properties such as optical absorption and emission from metal nanowire arrays in visible regime. We find that when *T* > 200 K, the OR spectrum for Ni/Cu superlattice nanowire array lies between those for Cu and Ni nanowire arrays. However, at lower temperatures (e.g., at 150 K), the intensity of the OR spectrum for Ni/Cu superlattice nanowire array is lower than those for Cu and Ni nanowire arrays.

**Figure 3:**
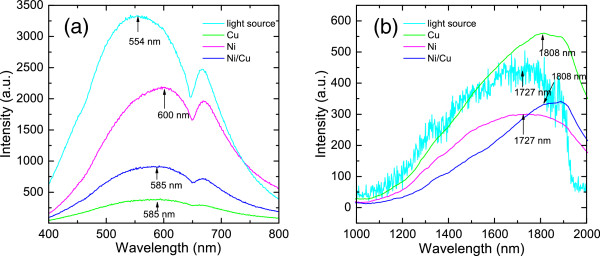
**The OR spectra for three kinds of nanowire arrays in visible**(**a**) **and near-infrared** (**b**)**bandwidths.** The measurements are carried out at room temperature. The intensity of the incident light source is shown as a reference. The peak positions are marked to guide the eye.

In Figure 3, the OR spectra are shown at room temperature for three metal nanowire array samples in visible and near-infrared bandwidths. In the visible regime (see Figure 3a), two relatively wide reflection peaks can be observed for all samples at about 500 to 650 nm and 650 to 700 nm, respectively. The 650- to 700-nm peaks for the three samples appear at almost the same position (at about 667 nm), while the 500- to 650-nm ones redshift slightly with respect to that of the incident light source. The peak position of the light source is at about 554 nm, whereas the peaks for Cu and Ni/Cu superlattice nanowire arrays are at about 585 nm and that for Ni nanowire arrays is at about 600 nm. It should be noted that the visible light source provided by the tungsten halogen lamp has two main peaks in the 400- to 800-nm wavelength regime. The intensity of infrared light source given by the Si carbide rod decreases when the radiation wavelength approaches 2 *μ*m. The variation of the intensity of the light sources is enhanced via measurement systems. We notice that Ni nanowire arrays reflect more strongly the visible light; Cu nanowire arrays reflect relatively weakly, and the OR spectrum for Ni/Cu superlattice nanowire arrays is just in between them. In the near-infrared range of 1,000 to 2,000 nm (see Figure 3b), the peaks of OR spectra for Cu nanowire arrays and Ni/Cu superlattice nanowire arrays are at about 1,808 nm, and Ni nanowire arrays and light source are at about 1,727 nm. The OR spectra for nanowire arrays redshift slightly with respect to the spectrum of the light source. In contrast to the visible regime, the Cu nanowire array reflects more strongly the infrared radiation than Ni nanowire array. Interestingly, the OR spectrum for Ni/Cu superlattice nanowire array is below that for Ni nanowire array when radiation wavelength is less than 1,730 nm, and it is located in between the OR spectra for Ni and Cu arrays when radiation wavelength is larger than 1,730 nm.

It is known that the OR spectrum of a metal nanostructure is determined mainly by surface plasmon modes and corresponding SPR. Our results indicate that the Cu, Ni, and Ni/Cu superlattice nanowire arrays show roughly the same OR spectra when the diameter and the length of the wires are the same. This implies that the features of the SPR in Ni, Cu, and Ni/Cu superlattice nanowire arrays have some similarities. From a fact that a strong optical reflection can weaken optical absorption and transmission, we can predict that Cu nanowire arrays can have stronger (weaker) optical absorption than Ni nanowire arrays in the visible (near-infrared) regime. The strong temperature dependence of the OR spectra for these array structures implies that there exists strong electron-phonon scattering in nanowire array samples. In the presence of light radiation field and phonon scattering, the electrons in an array structure can gain energy from the radiation field and lose energy via emission of phonons and excitation of plasmon and surface plasmon. At relatively low temperatures, the electron-phonon interaction is achieved mainly via phonon emission scattering channels, and the strength of the scattering increases with temperature. A strong phonon scattering implies a small electronic conductivity or a weak optical absorption and, thus, a strong OR. This is the main reason why the OR in metal nanowire arrays increases with temperature in the low-temperature regime. At relatively high temperatures, because phonon occupation number increases rapidly with temperature, the electron-phonon interaction is achieved not only through phonon emission, but also through phonon absorption. Phonon absorption can result in a gain of electron energy and in an increase in electronic conductivity. In this case, the effective strength of electron-phonon scattering decreases with increasing temperature, and therefore, the intensity of OR decreases with increasing temperature. It is interesting to note that such a mechanism is responsible to temperature-dependent electronic and optical properties in polar-semiconductor-based electronic systems. For example, it was found that the strongest magneto-phonon resonance can be observed at about 180 K for GaAs-based bulk and low-dimensional systems. However, we do not know the exact mechanism responsible to the decrease in OR for Cu nanowire arrays with increasing temperature when the temperature is within 4.2 to 70 K regime. This may suggest a strong metallic optic conduction in Cu nanowire array samples in this temperature regime.

We note that in a metal nanowire array, the visible (infrared) OR is caused mainly by SPR via interband (intraband) electronic transitions. Due to quantum confinement effect in the nanowire array structure, the surface plasmon and surface plasmon polariton modes induced by inter- and intraband transitions can have different features. For bulk metals, the interband SPR induced mainly by electronic transition from higher-energy *sp*-band to lower-energy *d*-band determines the color of the metal. At the same time, the intraband SPR within the *sp*- and *d*-bands gives free-carrier optic absorption which leads mainly to a lower-frequency background optic reflection. Because Cu is a better conductor than Ni, Ni normally reflects more strongly the visible light radiation than Cu does. However, for nanowire arrays, the electronic states in different bands are quantized. The intraband electronic transition accompanied by the absorption of photons can be achieved via inter-subband transition events which can result in resonant optical absorption when photon energy approaches the energy spacing between two subbands. Thus, intraband optical absorption can be enhanced in nanowire arrays. The results shown in Figure 3b indicate that the enhancement of intraband optical absorption in Ni nanowire arrays is stronger than that in Cu nanowire arrays. As a result, Cu nanowire arrays reflect more strongly the infrared radiation than Ni arrays do. Because the quantum confinement effect affects mainly the electronic states in different bands in the array structure, the main features of OR due to interband electronic transition does not change very significantly. This is why Ni nanowire arrays can reflect more strongly the visible radiation than Cu arrays can, as shown in Figure 3a and similar to the case for bulk materials.

Moreover, our results show that in the visible regime and when *T* > 200 K, the OR spectrum for Ni/Cu superlattice nanowire arrays lies between those for Cu and Ni nanowire arrays. However, at relatively lower temperatures (e.g., at 150 K), the intensity of the OR spectrum for Ni/Cu superlattice nanowire array is lower than those for Cu and Ni nanowire arrays. We believe that this may have resulted from different features of the phonon modes and electron-phonon scattering in nanowire and superlattice nanowire structures. In superlattice nanowire systems formed by different host materials, the phonon modes can be quantized and the conducting electrons are confined along the wire direction. The quantized phonon modes can weaken the electron-phonon scattering because a scattering event requires momentum and energy conservation. On the other hand, the localized electrons can interact more strongly with phonons. Our results suggest that when *T* > 200 K, the former case is dominant, and when *T* ≃ 150 K, the latter effect is stronger.

## Conclusions

In this study, Cu, Ni, and Ni/Cu nanowire arrays have been fabricated using state-of-the-art nanotechnology. The optical measurements on these nanowire arrays have been carried out in visible and near-infrared bandwidths for different temperatures. We have found that the optical reflection spectra of these samples depend strongly on temperature and on radiation wavelength. In particular, (1) the strongest OR in the visible regime can be observed at about 200 K for all samples, and (2) the OR for Cu nanowire arrays show a different dependence on temperature and radiation wavelength from that for Ni nanowire arrays. These results indicate that the surface plasmon resonances induced by inter- and intraband electronic transitions, the electron-phonon interaction, and the quantum confinement effect can play important roles in affecting optical properties of the metal nanowire array structure. We hope that the interesting experimental findings from this study can provide an in-depth understanding of optical properties of Cu and Ni nanowire arrays and Cu/Ni superlattice nanowire arrays and can provide a physical base for the application of metal nanowire arrays as advanced optical and optoelectronic devices.

## Competing interests

The authors declare that they have no competing interests.

## Authors’ contributions

WX proposed the research work, coordinated the collaboration, and carried out the analyses of experimental results. YYZ designed the experiment and experimental setup, carried out the measurements, and drafted the manuscript. SHX and GTF fabricated the nanowire and superlattice nanowire array samples. YMX and JGH participated in experimental measurements, results and discussion, and analyses. All authors read and approved the final manuscript.
